# Shallow reef fish assemblage from Fernando de Noronha Archipelago, Southwest Atlantic

**DOI:** 10.3897/BDJ.12.e133462

**Published:** 2024-12-03

**Authors:** Martina I. Ilarri, Leandro Valentim, Humberto M. Gitirana, Ricardo S. Rosa, Allan T. Souza

**Affiliations:** 1 Interdisciplinary Centre of Marine and Environmental Research (CIIMAR/CIMAR), University of Porto, Novo Edifício do Terminal de Cruzeiros do Porto de Leixões, Av. General Norton de Matos, s/n, 4450-208, Matosinhos, Portugal Interdisciplinary Centre of Marine and Environmental Research (CIIMAR/CIMAR), University of Porto, Novo Edifício do Terminal de Cruzeiros do Porto de Leixões, Av. General Norton de Matos, s/n, 4450-208 Matosinhos Portugal; 2 Diretoria de Licenciamento Ambiental, Instituto Brasileiro do Meio Ambiente e dos Recursos Naturais Renováveis - Ibama, Brasília, Brazil Diretoria de Licenciamento Ambiental, Instituto Brasileiro do Meio Ambiente e dos Recursos Naturais Renováveis - Ibama Brasília Brazil; 3 Universidade Estácio de Sá, Campus Nova Friburgo-RJ, Brasil, Rio de Janeiro, Brazil Universidade Estácio de Sá, Campus Nova Friburgo-RJ, Brasil Rio de Janeiro Brazil; 4 Programa de Pós-graduação em Ciências Biologicas, Universidade Federal da Paraíba, João Pessoa, Brazil Programa de Pós-graduação em Ciências Biologicas, Universidade Federal da Paraíba João Pessoa Brazil; 5 Institute for Atmospheric and Earth System Research INAR, Forest Sciences, Faculty of Agriculture and Forestry, P.O. Box 27, 00014 University of Helsinki, Helsinki, Finland, Helsinki, Finland Institute for Atmospheric and Earth System Research INAR, Forest Sciences, Faculty of Agriculture and Forestry, P.O. Box 27, 00014 University of Helsinki, Helsinki, Finland Helsinki Finland

**Keywords:** abundance, Brazil, endemic, oceanic island, *Stegastesrocasensis*, *Thalassomanoronhanum*

## Abstract

**Background:**

The paper presents an extensive dataset of the shallow reef fish communities and habitat characteristics in the Fernando de Noronha Archipelago (Southwest Atlantic). The data were collected from August to October 2006 in the Fernando de Noronha main island. To evaluate the shallow reef fish communities, 165 visual censuses were performed in eight different localities in the Fernando de Noronha Archipelago.

**New information:**

The dataset reports a comprehensive compilation of the shallow reef fish abundance, of eight localities of the Fernando de Noronha Archipelago. The dataset reveals spatial heterogeneity amongst the selected localities in terms of fish abundance, composition and size.

## Introduction

Oceanic islands are ecologically relevant environments because of their high biodiversity, which is characterised by a high degree of endemism (Kier et al. 2009). Their geographic isolation contributes to providing relevant information on the distribution, dispersal and establishment of different species. Reef fishes are an economically and ecologically important group that inhabits oceanic islands, occupying a wide range of ecological niches and serving as models to study relevant ecological interactions in these environments (Fernández-Cisternas et al. 2021). Since the mid-1990s, knowledge on the marine fishes and biogeographic patterns of islands in the Southwest Atlantic has steadily increased. To date, there were several studies published that focused on the ichthyofauna of Fernando de Noronha Archipelago. Most of them focused on species spatial distribution, animal behaviour and/or ecological interactions (Souza et al. 2011 , Pereira et al. 2012, [Bibr B11427261][Bibr B11427251], Bettcher et al. 2022) and only a few deal with new occurrences (Garla and Garcia 2008, Pimentel et al. 2020, Bucair et al. 2022, Mincarone et al. 2022), the description of a new species (Sampaio et al. 2004, Smith-Vaniz et al. 2018, Carvalho-Filho et al. 2020, Villarins et al. 2023), biogeography (Mendes 2007) and compilation work (Soto 1997, Soto 2001, Schmid et al. 2020, Salvetat et al. 2022).

Although the Archipelago has been the subject of several studies of ichthyofauna, only a few of these have aimed to characterise the structure of fish assemblage (Krajewski and Floeter 2011, Krajewski et al. 2011, Medeiros et al. 2011, Ilarri et al. 2017). Therefore, it is important to provide detailed information on shallow reef fish species for the different localities of the Archipelago of Fernando de Noronha.

From August to October 2006, the ichthyofauna of the shallow reef of Fernando de Noronha Archipelago was assessed daily through visual censuses in eight selected localities. The aim of this study is to report and make available the data on the abundance of the shallow reef fish species collected through visual censuses in Fernando de Noronha Archipelago. In this study, we provide an extensive list of reef fish occurrences from an area of remarkable importance for Atlantic reef fishes that is currently under-represented in large-scale ecological studies.

## Project description

### Study area description

**Figure 1.**
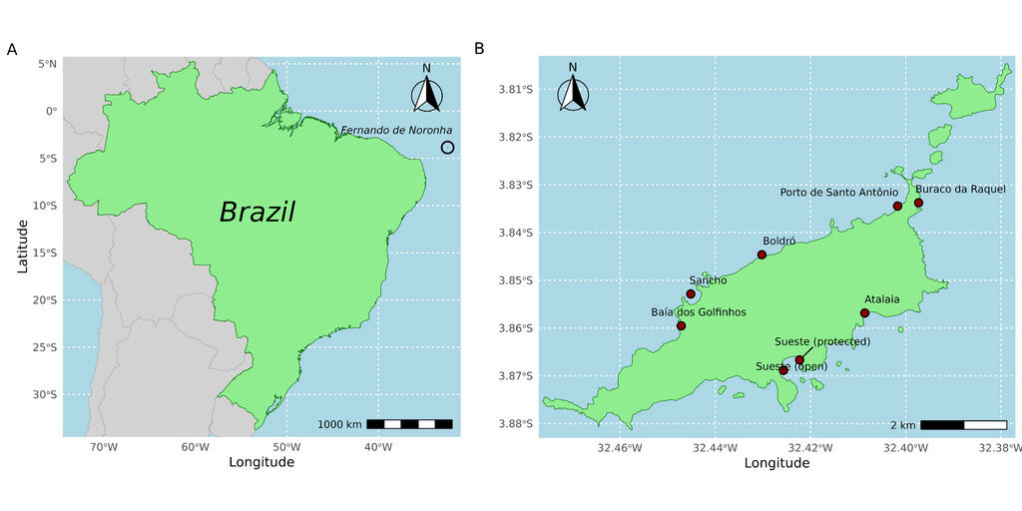
Map showing A) the location of the Fernando de Noronha Archipelago (black circle) in relation to Brazilian mainland and B) the eight sampled localities in the Archipelago (red dots).

The study was conducted in Atlantic Southwest, more specifically in the Fernando de Noronha Archipelago, an isolated group of volcanic islands, with one main island (https://deims.org/030bec0b-f6ac-4840-b226-af813258b14b) and 19 smaller adjacent islands and an area of 26 km^2^, located 345 km off the northeast coast of Brazil (3°54’S, 32°25’W) (Fig. 1).

Data collection was conducted at eight different localities within the Fernando de Noronha main island: Atalaia, Baía dos Golfinhos, Boldró, Buraco da Raquel, Porto de Santo Antônio, Sancho, Sueste (open) and Sueste (protected). These sites were chosen to cover the diversity of habitats in the Archipelago. Atalaia is a small reef lagoon with a predominance of sandy and rocky habitats. Baía dos Golfinhos is a calm and sheltered rocky area characterised by large pebbles and volcanic sand. Boldró comprises a reef flat area with sand and a rocky plateau in the intertidal zone and is an area exposed to currents. Buraco da Raquel comprises a reef lagoon characterised by sand and rocks mainly, exposed to currents. Porto de Santo António is characterised by a reef area with sandy and rocky habitats mainly and is a calm and sheltered area. Sancho comprises a bay with sand and large pebbles and rocks and is a calm and sheltered area. Sueste (open) is a bay with rock and sand that has a central plateau and is a calm and sheltered area. Sueste (protected) is a bay with rocks, sand and pebbles and is a calm and sheltered area.

### Funding

Financial support was provided by CAPES (Coordenação de Aperfeiçoamento de Pessoal de Nível Superior) to the first author and by the Graduate Program in Biological Science (Zoology) of Universidade Federal da Paraíba. Martina I. Ilarri is currently supported by national funds through FCT – Foundation for Science and Technology, Portugal, within the scope of UIDB/04423/2020 and UIDP/04423/2020 and a research contract (DL57/2016/CP1344/CT0018). A. T. Souza is funded by eLTER PLUS (European Union's Horizon 2020 Research and Innovation Programme under grant agreement No 871128) and BioDT (https://doi.org/10.3030/101057437).

## Sampling methods

### Study extent

Study extent

### Sampling description

**Figure 2.**
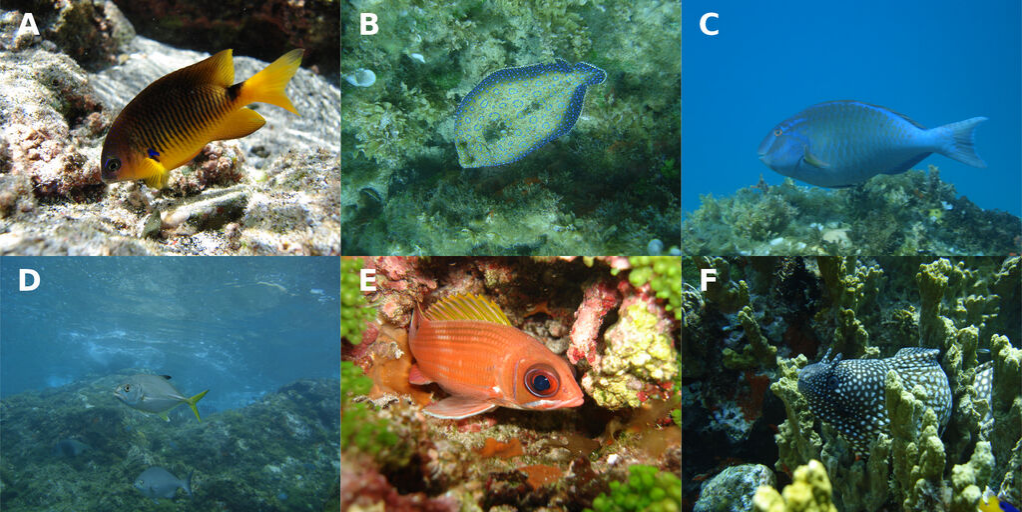
A plate with representative reef fish species observed during the visual censuses in the Fernando de Noronha Archipelago, Southwest Atlantic. **A** *Stegastesrocasensis*; **B** *Bothuslunatus*; **C** *Sparisomafrondosum*; **D** *Caranxlatus*; **E** *Holocentrusadscensionis*; **F** *Muraenapavonina*. Photos by Allan T. Souza.

To assess the shallow reef fish communities, a total of 165 visual censuses were performed from August to October 2006 (with a minimum of 20 censuses per locality). All observations were made by free diving in areas with depths up to six metres (m) during the day (from 0800 to 1800 h). To account for possible tidal and temporal influences, observations were distributed throughout the day (morning and afternoon) and different tidal regimes (ebb and flood). The fish assemblage was assessed using a belt-transect (30 m x 2 m), based on the belt-transect visual census method (Brock 1954). All censuses were made by the same diver, swimming at a constant speed, having previously stood by for five minutes to minimise disturbance caused by the diver's presence (Russ 1989). Fishes were identified according to Humann and Deloach (2002a), Carvalho-Filho (2023) and Floeter et al. (2023), counted and had their size visually estimated to the nearest centimetre (Fig. 2). Sampling was conducted randomly in selected areas of eight localities with predominantly (75%) consolidated substrate (Luckhurst and Luckhurst 1978, Ohlhorst et al. 1988, Littler et al. 1989, Rogers et al. 1994, Humann and Deloach 2002b).

### Quality control

While visual censuses are often used to estimate fish populations on reefs, they have several limitations (Harvey et al. 2004, Bernard et al. 2013). Observer bias and varying skill levels can affect accuracy and lead to inconsistent data. Fish behaviour, such as avoiding divers or hiding, skews counts, especially for shy or nocturnal species, can vary significantly. Water clarity and light conditions also affect visibility and identification. In addition, visual censuses usually only cover small areas, so wider population trends may be missed. All of these factors limit the reliability and representativeness of visual censuses for the comprehensive assessments of fish populations on reefs and, therefore, these known issues must be taken into account when using this dataset. On the other hand, visual censuses have the advantage that they are non-lethal, providing instant access to the data on species and are also cheaper and easier to implement than other sampling methods.

## Geographic coverage

### Description

This study was carried out in eight different localities (Atalaia, Baía dos Golfinhos, Boldró, Buraco da Raquel, Porto de Santo Antônio, Sancho, Sueste (open), Sueste (protected) within the Archipelago of Fernando de Noronha, Southwest Atlantic, Brazil (Fig. 1).

### Coordinates

-3.86886 and -3.83987 Latitude; -32.44712 and -32.40372 Longitude.

## Taxonomic coverage

### Description

**Figure 3.**
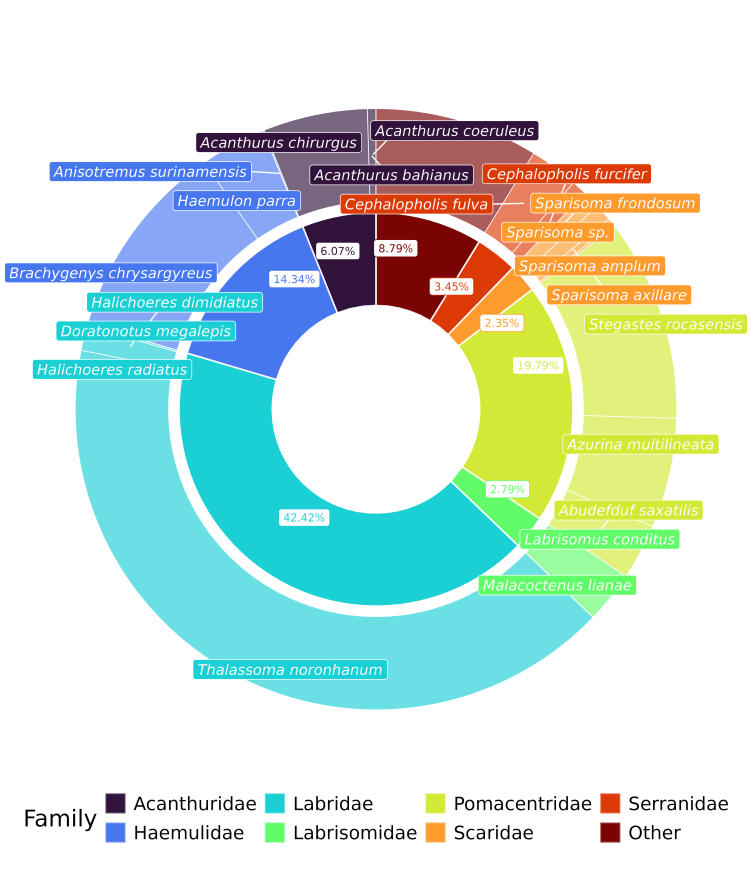
Sunburst plot showing the proportion of the most representative species per family observed in the visual censuses made in the Fernando de Noronha Archipelago, Southwest Atlantic. The inner circle shows the percentage of individuals recorded by families, while the outer circle displays the proportional abundance of the most representative species recorded in this study. The seven most abundant fish families are displayed individually, while the remainder families are pooled together in the category named Other which contains fishes from 22 families (Aulostomidae, Balistidae, Belonidae, Blennidae, Bothidae, Carangidae, Carcharhinidae, Cheatodontidae, Clupeidae, Dasyatidae, Gobiidae, Hemiramphidae, Holocentridae, Kyphosidae, Lutjanidae, Monacanthidae, Mullidae, Muraenidae, Ophichthidae, Ostraciidae, Pomacanthidae and Sphyraenidae) and 32 species (*Acanthostracionpolygonium*, *Aluterusscriptus*, *Aulostomusstrigosus*, *Bothuslunatus*, *Cantherhinespullus*, *Carangoidesbartholomaei*, *Caranxcrysos*, *C.latus*, *C.lugubris*, *Chaetodonocellatus*, *Coryphopterusglaucofraenum*, *Echidnacatenata*, *Gymnothoraxmiliaris*, *Harengulajaguana*, *Hemiramphusbrasiliensis*, *Holocentrusadscensionis*, *Hypanusberthalutzae*, *Kyphosus* sp., *Lactophrystrigonus*, *Lutjanusjocu*, *Melichthysniger*, *Mulloidichthysmartinicus*, *Muraenapavonina*, *Myrichthysocellatus*, *Myripristisjacobus*, *Negaprionbrevirostris*, *Ophioblenniustrinitatis*, *Platybeloneargalus*, *Pomacanthusparu*, *Pseudupeneusmaculatus*, *Sphyraenabarracuda* and *Sphyraenaguachancho*).

**Figure 4.**
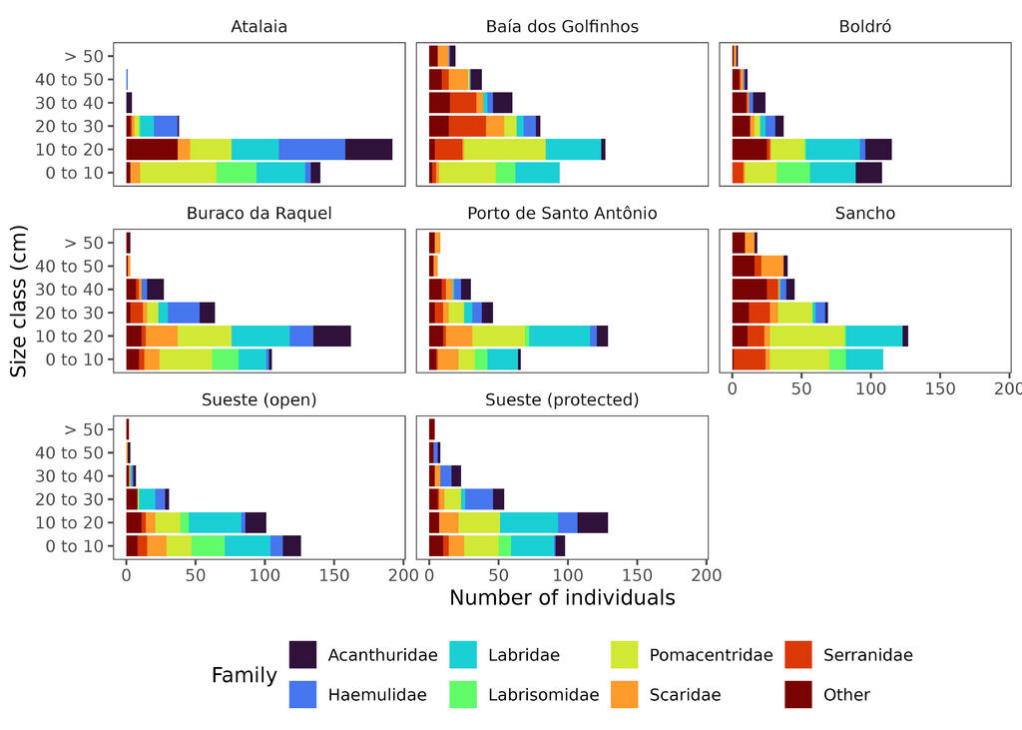
Size class (0-10; 10-20; 20-30; 30-40; 40-50; > 50 cm) per study area of the most representative fish families observed in the visual censuses made in the Fernando de Noronha Archipelago, SW Atlantic. Less representative families were pooled together and were displayed as Other in the graph. The Other category contains fishes from 22 families (Aulostomidae, Balistidae, Belonidae, Blennidae, Bothidae, Carangidae, Carcharhinidae, Cheatodontidae, Clupeidae, Dasyatidae, Gobiidae, Hemiramphidae, Holocentridae, Kyphosidae, Lutjanidae, Monacanthidae, Mullidae, Muraenidae, Ophichthidae, Ostraciidae, Pomacanthidae and Sphyraenidae) and 32 species (*Acanthostracionpolygonium*, *Aluterusscriptus*, *Aulostomusstrigosus*, *Bothuslunatus*, *Cantherhinespullus*, *Carangoidesbartholomaei*, *Caranxcrysos*, *C.latus*, *C.lugubris*, *Chaetodonocellatus*, *Coryphopterusglaucofraenum*, *Echidnacatenata*, *Gymnothoraxmiliaris*, *Harengulajaguana*, *Hemiramphusbrasiliensis*, *Holocentrusadscensionis*, *Hypanusberthalutzae*, *Kyphosus* sp., *Lactophrystrigonus*, *Lutjanusjocu*, *Melichthysniger*, *Mulloidichthysmartinicus*, *Muraenapavonina*, *Myrichthysocellatus*, *Myripristisjacobus*, *Negaprionbrevirostris*, *Ophioblenniustrinitatis*, *Platybeloneargalus*, *Pomacanthusparu*, *Pseudupeneusmaculatus*, *Sphyraenabarracuda* and *Sphyraenaguachancho*).

The dataset contains the records of 15,065 individuals belonging to 51 species and two unidentified species, from 29 families (Fig. 3) with *Thalassomanoronhanum* and *Stegastesrocasensis* representing the vast majority of the individuals (Fig. 3). On average, the largest individuals were recorded at Baía dos Golfinhos and Sancho, while Atalaia and Buraco da Raquel made the largest contribution of small fish individuals (Fig. 4). The taxa identification numbers (acceptedNameUsageID) were based on GBIF Backbone Taxonomy (GBIF Secretariat 2022). The scientific names of the taxa, their authorship and year and original descriptions followed Fricke et al. (2024), whereas the common name of the species was based on FishBase (Froese and Pauly 2024).

### Taxa included

**Table:** 

Rank	Scientific Name	Common Name
species	*Abudefdufsaxatilis* (Linnaeus, 1758)	Sergeant-major
species	*Acanthostracionpolygonium* Poey, 1876	Honeycomb cowfish
species	*Acanthurusbahianus* Castelnau, 1855	Barber surgeonfish
species	*Acanthuruschirurgus* (Bloch, 1787)	Doctorfish
species	*Acanthuruscoeruleus* Bloch & Schneider, 1801	Blue tang surgeonfish
species	*Aluterusscriptus* (Osbeck, 1765)	Scribbled leatherjacket filefish
species	*Anisotremussurinamensis* (Bloch, 1791)	Black margate
species	*Aulostomusstrigosus* Wheeler, 1955	Trumpetfish
species	*Azurinamultilineata* (Guichenot, 1853)	Brown chromis
species	*Bothuslunatus* (Linnaeus, 1758)	Plate fish
species	*Brachygenyschrysargyreus* (Günther, 1859)	Smallmouth grunt
species	*Cantherhinespullus* (Ranzani, 1842)	Orangespotted filefish
species	*Carangoidesbartholomaei* (Cuvier, 1833)	Yellow jack
species	*Caranxcrysos* (Mitchill, 1815)	Blue runner
species	*Caranxlatus* Agassiz, 1831	Horse-eye jack
species	*Caranxlugubris* Poey, 1860	Black jack
species	*Cephalopholisfulva* (Linnaeus, 1758)	Coney
species	*Cephalopholisfurcifer* (Valenciennes, 1828)	Creole-fish
species	*Chaetodonocellatus* Bloch, 1787	Spotfin butterflyfish
species	*Coryphopterusglaucofraenum* Gill, 1863	Bridled goby
species	*Doratonotusmegalepis* Günther, 1862	Dwarf wrasse
species	*Echidnacatenata* (Bloch, 1795)	Chain moray
species	*Gymnothoraxmiliaris* (Kaup, 1856)	Goldentail moray
species	*Haemulonparra* (Desmarest, 1823)	Sailor's grunt
species	*Halichoeresdimidiatus* (Agassiz, 1831)	
species	*Halichoeresradiatus* (Linnaeus, 1758)	Puddingwife wrasse
species	*Harengulajaguana* Poey, 1865	Scaled herring
species	*Hemiramphusbrasiliensis* (Linnaeus, 1758)	Ballyhoo halfbeak
species	*Holocentrusadscensionis* (Osbeck, 1765)	Squirrelfish
species	*Hypanusberthalutzae* Petean, Naylor & Lima, 2020	Lutz's stingray
genus	*Kyphosus* Lacepède, 1801	
species	*Labrisomusconditus* Sazima, Carvalho-Filho, Gasparini & Sazima, 2009	Masquerader hairy blenny
species	*Lactophrystrigonus* (Linnaeus, 1758)	Buffalo trunkfish
species	*Lutjanusjocu* (Bloch & Schneider, 1801)	Dog snapper
species	*Malacoctenuslianae* Carvalho-Filho, Almeida, Britto, Dias & Lima, 2020	Saddled blenny
species	*Melichthysniger* (Bloch, 1786)	Black triggerfish
species	*Mulloidichthysmartinicus* (Cuvier, 1829)	Yellow goatfish
species	*Muraenapavonina* Richardson, 1845	Whitespot moray
species	*Myrichthysocellatus* (Lesueur, 1825)	Goldspotted eel
species	*Myripristisjacobus* Cuvier, 1829	Blackbar soldierfish
species	*Negaprionbrevirostris* (Poey, 1868)	Lemon shark
species	*Ophioblenniustrinitatis* Miranda Ribeiro, 1919	
species	*Platybeloneargalus* (Lesueur, 1821)	Keeltail needlefish
species	*Pomacanthusparu* (Bloch, 1787)	French angelfish
species	*Pseudupeneusmaculatus* (Bloch, 1793)	Spotted goatfish
species	*Sparisomaamplum* (Ranzani, 1841)	Reef parrotfish
species	*Sparisomaaxillare* (Steindachner, 1878)	Gray parrotfish
species	*Sparisomafrondosum* (Agassiz, 1831)	Agassiz's parrotfish
genus	*Sparisoma* Swainson, 1839	
species	*Sphyraenabarracuda* (Edwards, 1771)	Great barracuda
species	*Sphyraenaguachancho* Cuvier, 1829	Guachanche barracuda
species	*Stegastesrocasensis* (Emery, 1972)	Rocas gregory
species	*Thalassomanoronhanum* (Boulenger, 1890)	Noronha wrasse

## Temporal coverage

**Data range:** 2006-8-28 – 2006-10-28.

## Usage licence

### Usage licence

Other

### IP rights notes

**CC BY 4.0**

## Data resources

### Data package title

fdd-reef-fish

### Resource link

https://ipt.pensoft.net/manage/resource.do?r=fdd-reef-fish

### Number of data sets

2

### Data set 1.

#### Data set name

event

#### Data format

txt

#### Download URL

https://ipt.pensoft.net/archive.do?r=fdd-reef-fish&v=1.3

#### Description

The event dataset includes 17 terms that follow the Darwin Core standard (Darwin Core Maintenance Group 2021) whenever possible. The dataset contains 165 events, with Atalaia, Baía dos Golfinhos, Buraco da Raquel, Porto de Santo Antônio and Sueste (open) having 20 events in each, while Boldró and Sueste (protected) had 21 events in each, with Sancho having 23 events. Events ranged from 28 August to 28 October 2006, with the earliest events starting at 07:55 h and the latest event starting at 17:15 h.

**Data set 1.** 

Column label	Column description
eventID	An identifier for the set of information associated with a dwc:Event (something that occurs at a place and time). May be a global unique identifier or an identifier specific to the dataset.
eventDate	The date-time or interval during which a dwc:Event occurred. For occurrences, this is the date-time when the dwc:Event was recorded. Not suitable for a time in a geological context.
eventTime	The time or interval during which a dwc:Event occurred.
startDayOfYear	The earliest integer day of the year on which the dwc:Event occurred (1 for 1 January, 365 for 31 December, except in a leap year, in which case it is 366).
country	The name of the country or major administrative unit in which the dcterms:Location occurs.
countryCode	The standard code for the country in which the dcterms:Location occurs.
locality	The specific description of the place.
locationID	An identifier for the set of dcterms:Location information. May be a global unique identifier or an identifier specific to the dataset.
geodeticDatum	The ellipsoid, geodetic datum or spatial reference system (SRS) upon which the geographic coordinates given in dwc:decimalLatitude and dwc:decimalLongitude are based.
coordinateUncertaintyInMetres	The horizontal distance (in metres) from the given dwc:decimalLatitude and dwc:decimalLongitude describing the smallest circle containing the whole of the dcterms:Location. Leave the value empty if the uncertainty is unknown, cannot be estimated or is not applicable (because there are no coordinates). Zero is not a valid value for this term.
decimalLatitude	The geographic latitude (in decimal degrees, using the spatial reference system given in dwc:geodeticDatum) of the geographic centre of a dcterms:Location. Positive values are north of the Equator, negative values are south of it. Legal values lie between -90 and 90, inclusive.
decimalLongitude	The geographic longitude (in decimal degrees, using the spatial reference system given in dwc:geodeticDatum) of the geographic centre of a dcterms:Location. Positive values are east of the Greenwich Meridian, negative values are west of it. Legal values lie between -180 and 180, inclusive.
samplingProtocol	The names of, references to, or descriptions of the methods or protocols used during a dwc:Event.
samplingEffort	The amount of effort expended during a dwc:Event.
sampleSizeValue	A numeric value for a measurement of the size (time duration, length, area or volume) of a sample in a sampling dwc:Event.
sampleSizeUnit	The unit of measurement of the size (time duration, length, area or volume) of a sample in a sampling dwc:Event.
recordedBy	A list (concatenated and separated) of names of people, groups or organisations responsible for recording the original dwc:Occurrence. The primary collector or observer, especially one who applies a personal identifier (dwc:recordNumber), should be listed first.

### Data set 2.

#### Data set name

occurrence

#### Data format

txt

#### Download URL

https://ipt.pensoft.net/archive.do?r=fdd-reef-fish&v=1.3

#### Description

The occurrence dataset includes 21 terms that follow the Darwin Core standard (Darwin Core Maintenance Group 2021) whenever possible. The dataset contains 2735 observations, from 53 different taxonomic entities (51 species and 2 genera). A total of 15065 individuals were recorded, ranging from 3 to 130 cm of Total Length (TL).

**Data set 2.** 

Column label	Column description
eventID	An identifier for the set of information associated with a dwc:Event (something that occurs at a place and time). May be a global unique identifier or an identifier specific to the dataset.
ownerInstitutionCode	The name (or acronym) in use by the institution having ownership of the object(s) or information referred to in the record.
basisOfRecord	The specific nature of the data record.
occurrenceID	An identifier for the dwc:Occurrence (as opposed to a particular digital record of the dwc:Occurrence). In the absence of a persistent global unique identifier, construct one from a combination of identifiers in the record that will most closely make the dwc:occurrenceID globally unique.
occurrenceStatus	For dwc:Occurrences, the default vocabulary is recommended to consist of present and absent, but can be extended by implementers with good justification. This term has an equivalent in the dwciri: namespace that allows only an IRI as a value, whereas this term allows for any string literal value.
kingdom	The full scientific name of the kingdom in which the dwc:Taxon is classified.
phylum	The full scientific name of the phylum or division in which the dwc:Taxon is classified.
order	The full scientific name of the order in which the dwc:Taxon is classified.
family	The full scientific name of the family in which the dwc:Taxon is classified.
genus	The full scientific name of the genus in which the dwc:Taxon is classified.
specificEpithet	The name of the first or species epithet of the dwc:scientificName.
scientificName	The full scientific name, with authorship and date information if known. When forming part of a dwc:Identification, this should be the name in lowest level taxonomic rank that can be determined. This term should not contain identification qualifications, which should instead be supplied in the dwc:identificationQualifier term.
establishmentMeans	Statement about whether a dwc:Organism has been introduced to a given place and time through the direct or indirect activity of modern humans.
taxonRank	The taxonomic rank of the most specific name in the dwc:scientificName.
taxonID	A global unique identifier for the taxon (name in a classification).
identifiedBy	A list (concatenated and separated) of names of people, groups or organisations who assigned the dwc:Taxon to the subject.
dateIdentified	The date on which the subject was determined as representing the dwc:Taxon.
identificationReferences	A list (concatenated and separated) of references (publication, global unique identifier, URI) used in the dwc:Identification.
organismQuantity	A number or enumeration value for the quantity of dwc:Organisms.
organismQuantityType	The type of quantification system used for the quantity of dwc:Organisms.
dynamicProperties	A list of additional measurements, facts, characteristics or assertions about the record. Meant to provide a mechanism for structured content.

## Additional information

This work was carried out in accordance with Brazilian legal requirements, including those related to the conservation and protection of animals.
